# Regional changes in cerebral perfusion with age when accounting for changes in gray‐matter volume

**DOI:** 10.1002/mrm.30376

**Published:** 2024-11-20

**Authors:** Jian Hu, Martin S. Craig, Silvin P. Knight, Celine De Looze, James F. Meaney, Rose Anne Kenny, Xin Chen, Michael A. Chappell

**Affiliations:** ^1^ Mental Health & Clinical Neurosciences, School of Medicine University of Nottingham Nottingham UK; ^2^ Sir Peter Mansfield Imaging Center, School of Medicine University of Nottingham Nottingham UK; ^3^ The Irish Longitudinal Study on Ageing, School of Medicine Trinity College Dublin Dublin Ireland; ^4^ School of Medicine Trinity College Dublin Dublin Ireland; ^5^ The National Center for Advanced Medical Imaging St. James's Hospital Dublin Ireland; ^6^ The Global Brain Health Institute Trinity College Dublin Dublin Ireland; ^7^ Mercer's Institute for Successful Ageing St. James's Hospital Dublin Ireland; ^8^ Intelligent Modelling & Analysis Group School of Computer Science, University of Nottingham Nottingham UK

**Keywords:** arterial spin labeling, brain atrophy, partial volume effects correction, perfusion, surface‐based analysis

## Abstract

**Purpose:**

One possible contributing factor for cerebral blood flow (CBF) decline in normal aging is the increase in partial volume effects due to brain atrophy, as cortical thinning can exacerbate the contamination of gray‐matter (GM) voxels by other tissue types. This work investigates CBF changes in normal aging of a large elderly cohort aged 54 to 84 and how correction for partial volume effects that would accommodate potential changes in GM might affect this.

**Methods:**

The study cohort consisted of 474 participants aged 54 to 84 years using pseudo‐continuous arterial spin labeling MRI. A volumetric pipeline and a surface‐based pipeline were applied to measure global and regional perfusion. Volumetric regions of interest (ROIs) included GM, cerebral white matter, vascular territories, and the brain atlas from the UK Biobank. The cortical parcellation was using Desikan–Killiany atlas. Non–partial volume effect correction (PVEc) and PVEc GM‐CBF changes with aging were modeled using linear regressions.

**Results:**

Global GM CBF decreased by 0.17 mL/100 g/min per year with aging before PVEc (*p* < 0.05) and was 0.18 mL/100 g/min after PVEc (*p* < 0.05). All cortical parcels exhibited CBF decreases with age before PVEc. After PVEc, seven parcels retained decreasing trends. However, GM CBF demonstrated increase with age after PVEc in three parcels.

**Conclusion:**

Although decreases in global perfusion are observed with aging before PVEc, perfusion variations appear to be more regionally selective after PVEc. This supports the understanding that variation in cerebral perfusion with age observed with imaging is influenced by regional changes in anatomy that can be accommodated with PVEc, but perfusion variation is still observable even after PVE is accounted for.

## 1 INTRODUCTION

Perfusion is the process of delivering blood to capillaries in tissues, which is critical for maintaining the health and function of tissues and organs by supplying oxygen and nutrients while removing metabolic waste products.^1^, ^2^ Cerebral blood flow (CBF) refers to the rate of delivery of arterial blood through the capillaries and is closely linked to the normal metabolism. Arterial spin labeling (ASL) MRI provides the only noninvasive way to image CBF in the brain using blood water as an endogenous tracer.^3^, ^4^ Briefly, ASL uses radiofrequency pulses to invert the magnetization of arterial blood water protons for labeling.^5^ The effect of labeling can be obtained by subtracting a labeled image from a non‐labeled (control) image. A perfusion‐weighted image can result from averaging the difference of label‐control image pairs. To quantify CBF, a separate proton density–weighted image is required to calibrate the perfusion‐weighted image.^6^ ASL has become a prevalent method for examining CBF in different neurological conditions, as well as for studying the influences of age and gender effects. Although there is a consensus that CBF tends to decrease with age increasing, findings are varied when it comes to the impact of age and gender on CBF in specific brain regions. For instance, some research points to a decline in cortical CBF with advancing age; however, other studies suggest an increase or no significant correlation with age.^7^, ^8^, ^9^, ^10^, ^11^, ^12^ These discrepancies are likely due to limited cohort sizes and inconsistencies in the analysis methods used.

Like other perfusion techniques, ASL has a relatively low spatial resolution typically with voxel size around 3–5 mm. This resolution is lower than the anatomical variations within the tissues it aims to image. This can significantly affect CBF measurements affected by partial volume effects (PVEs), as a voxel is likely to contain more than one type of tissue. Although this might not be evidently problematic when visually examining areas of hyperperfusion or hypoperfusion, such as brain tumors or stroke, PVEs can confuse the quantitative measurements of CBF, especially when comparing individuals or detecting subtle perfusion changes. This is particularly relevant in cases of neurodegenerative conditions, such as tissue atrophy,^13^ or certain stroke effects. In these scenarios, actual changes in blood flow might be misinterpreted for, or masked by CBF differences or changes between corresponding voxels in the same anatomical voxels. It is increasingly recognized that correcting PVE is crucial to quantify gray‐matter (GM) CBF independently of any confounding effects from partial volumes (PVs) of white matter (WM) or cerebral spinal fluid (CSF).^14^, ^15^, ^16^ Therefore, various partial volume effects correction (PVEc) methods have been developed that use voxel‐wise estimates of PVs to identify the signal from each tissue. Examples include the Muller‐Gartner method for positron emission tomography,^17^ and linear regression^18^ or spatially regularized variational Bayes methods for ASL.^14^

Traditional methods for obtaining PV estimates rely on volumetric segmentation. Such methods may not provide precise results for intricate structures like the cortex, with its efficacy being contingent on the accuracy of the segmentation method used. Given the complexity of shapes such as the thin, intricately folded cerebral cortex, surface‐based segmentation has become increasingly popular, especially with tools like *FreeSurfer*.^19^ This method offers significant advantages. First, it allows for more continuous representation because it places surface vertices with precision finer than a voxel, in contrast to the inherently discrete nature of volumetric segmentation. Second, it enables the application of anatomically accurate constraints that vary directionally, such as ensuring tissue homogeneity along a surface while allowing for heterogeneity perpendicular to it. Although there are surface‐based PV estimation tools in existing studies, previous attempts have typically been tailored for a particular imaging modality, such as the Human Connectome Project's (HCP) functional MRI surface pipeline for BOLD (blood oxygen level dependent) using the ribbon‐constrained method^20^ and *PETSurfer*,^21^ a variant of *FreeSurfer*, for positron emission tomography.

Current brain research using ASL predominantly relies on region‐of‐interest (ROI) analysis or volumetric (i.e., voxel‐based) analysis. In the ROI approach, researchers define anatomical or functional brain regions and compute a quantity of interest within these regions. This method, however, is limited by the prior hypotheses to determine the expected ROI. On the other hand, the volumetric method has an advantage in identifying unanticipated or non‐hypothesized areas of abnormal activity in the brain.^22^ Surface‐based analysis has become increasingly popular in neuroimaging because of its effectiveness in representing the cortex.^22^

Some studies have used cortical surface–based analysis in perfusion to investigate regional CBF changing with aging.^22^, ^23^, ^24^, ^25^ However, few studies use cortical surface–based analysis with ASL data.^7^, ^26^ A prior study concluded that performing cortical surface–based analysis of ASL is technically achievable, yielding high‐quality images, and it has the potential to markedly enhance the identification of focal perfusion changes in neurodegenerative diseases in a clinical setting.^27^

**FIGURE 1:**
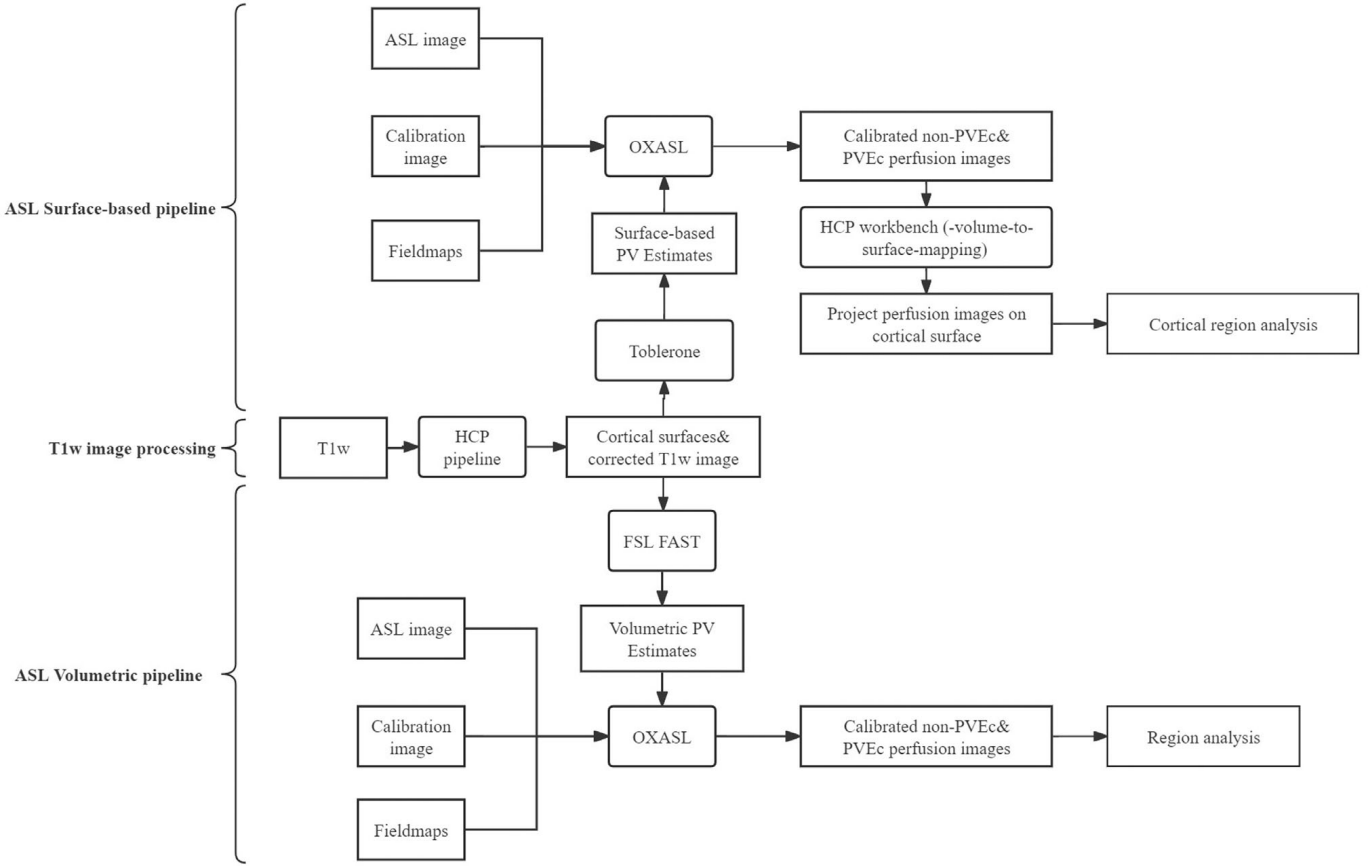
*Top and bottom*: Arterial spin labeling (ASL) surface‐based pipeline and ASL volumetric pipelines, respectively. *Middle*: T_1_‐weighted (T1w) processing pipeline. HCP, Human Connectome Project; PV partial volume; PVEc, partial volume effect correction.

A previous study focusing on the elderly cohort from TILDA (The Irish Longitudinal Study on Aging) ASL data^28^ investigated age‐related perfusion changes and reported on mean global brain perfusion. The objective of this study was to perform a subsequent exploratory investigation of the influence of aging on regional brain perfusion and the potential influence of changes in anatomy on these measures in the same elderly data set with and without PV correction. This study uses a conventional volumetric representation but also explores the same relationships using a surface‐based representation that is increasingly becoming widely used for functional brain imaging studies. Consequently, two distinct pipelines, one volumetric and the other surface‐based, were applied concurrently (Figure 1). In the volumetric pipeline, PV estimates were obtained through FSL FAST,^29^ and subsequent analysis for subcortex were performed at the voxel level. Meanwhile, a surface‐based pipeline was developed for cortex analysis using PV estimates from Toblerone.^30^ PVEs were corrected in this study using spatially regularized variational Bayes for ASL.^14^

## 2 METHODS

### 2.1 Data set

This study used a subset of participants from TILDA, a prospective cohort study that collects health, economic, and social aspects of Irish adults.^31^, ^32^ The data set consisted of 474 subjects with available T_1_‐weighted and pseudo‐continuous ASL (pCASL) images. The exclusion criteria were detailed in the prior study.^28^ Briefly, participants were excluded if they presented any contraindications to MRI, a history of stroke or head injury, or if their images contained artifacts. Additionally, subjects outside the specified age range of 54 to 84 years were excluded. Importantly, participants with extreme mean GM CBF values in non‐PVEc data (< 10 or > 100 mL/100 g/min) were also excluded from the study of perfusion changes with age. This exclusion criterion led to a discrepancy in the total number of participants included in this study compared with the prior study^28^ due to different calibration methods resulting in differences of perfusion estimates. Consequently, the final data set in this study consisted of 423 healthy participants (215 females and 208 males) aged 54 to 84 years. The characteristics of the final data set are presented in Table 1, including age, sex, education, and health conditions.

**TABLE 1:** Characteristics of the data set used in this study.

	Total subjects = 423
Age (years)	
Male	67.7 (SD: 6.5, range: 54–84)
Female	67.8 (SD: 7.0, range: 54–84)
Sex (% [*n*])	Female: 50.8% (215)
Education (% [*n*])	
Primary/none	20.6% (87)
Secondary	36.6% (155)
Third/higher	42.8% (181)
Mean BMI (kg/m^2^)	27.9 (SD: 4.2, range: 18.1–45.8)
Number of cardiovascular conditions (% [*n*])	
0	38.3% (162)
1	32.9% (139)
2+	28.8% (122)
High blood pressure (% [*n*])	35.2% (149)
Smoker (% [*n*])	
Never	51.5% (218)
Past	41.6% (176)
Current	6.9% (29)
CAGE alcohol scale (% [*n*])	
0	79.9% (338)
1	8.7% (37)
No response	11.3% (48)
Mean MMSE	28.9 (SD: 1.3, range: 24–30)

*Note*: Cardiovascular conditions included high cholesterol, high blood pressure, angina, heart attack ever, heart failure, murmur, abnormal rhythm, stroke ever, and transient ischemic attack ever.

Abbreviations: BMI, body mass index; MMSE, Mini‐Mental State Examination; SD, standard deviation.

### 2.2 MRI data acquisition

T_1_‐weighted and pCASL sequences were acquired on a 3T system using a 32‐channel head coil. T_1_‐weighted three‐dimensional magnetization‐prepared rapid gradient‐echo anatomical images were acquired over 5 min 24 s with the following scan parameters: field of view = 240 × 240 × 162 mm^3^, matrix = 288 × 288 × 180, repetition time = 6.7 ms, echo time = 3.1 ms, flip angle = 8°, and sensitivity encoding = 2. pCASL acquisition parameters were as follows: 30 interleaved pairs of images acquired alternating with and without ASL, field of view = 240 × 240 mm^2^, matrix = 80 × 80, repetition time = 4000 ms, echo time = 9 ms, flip angle = 90°, sensitivity encoding = 2.5, and scan duration = 4 min 16 s. Thirteen slices (8‐mm thick, 1‐mm gap) were acquired sequentially in a caudocranial direction. A labeling duration of 1800 ms and a post‐label delay (PLD) of 1800 ms were used. Calibration scans measuring the equilibrium magnetization (M_0_) were also acquired using the same geometry as the pCASL sequence, with TR = 10 000 ms, echo time = 9 ms, and scan duration = 20 s. B_0_ field maps were measured using a two‐echo two‐dimensional gradient‐echo sequence with the same in‐plane resolution as the pCASL scans and the following acquisition parameters: repetition time = 455 ms, echo time 1/echo time 2 = 1.69/7.0 ms, flip angle = 90°, and scan duration = 39 s.

### 2.3 Ethics statement

All research was conducted in accordance with the Declaration of Helsinki. Ethical approval for each wave of TILDA was granted by the Health Sciences Research Ethics Committee at Trinity College Dublin (Dublin, Ireland), and all participants provided written informed consent. Participants undergoing MRI were required to complete an additional MRI‐specific consent form, with further ethical approval obtained from the St James's Hospital Research Ethics Committee (Dublin, Ireland).

### 2.4 Data processing

#### 2.4.1 Structural image processing

T_1_‐weighted images were processed using the HCP minimal processing pipelines,^20^, ^33^ which consist of three distinct stages: (1) the *PreFreeSurfer* pipeline, which corrects for gradient distortions and bias fields (B_1_ inhomogeneities), performs brain extraction, and aligns the images to a standard reference space; (2) the *FreeSurfer* pipeline, which segments structural volumes according to a specified parcellation, reconstructs white and pial cortical surfaces, and registers these surfaces to the *FreeSurfer* surface atlas; and (3) the *PostFreeSurfer* pipeline, which generates the final NIFTI files and GIFTI surface files registered to the Conte69 surface atlas.^20^, ^34^

#### 2.4.2 Partial volume estimation

**FIGURE 2:**
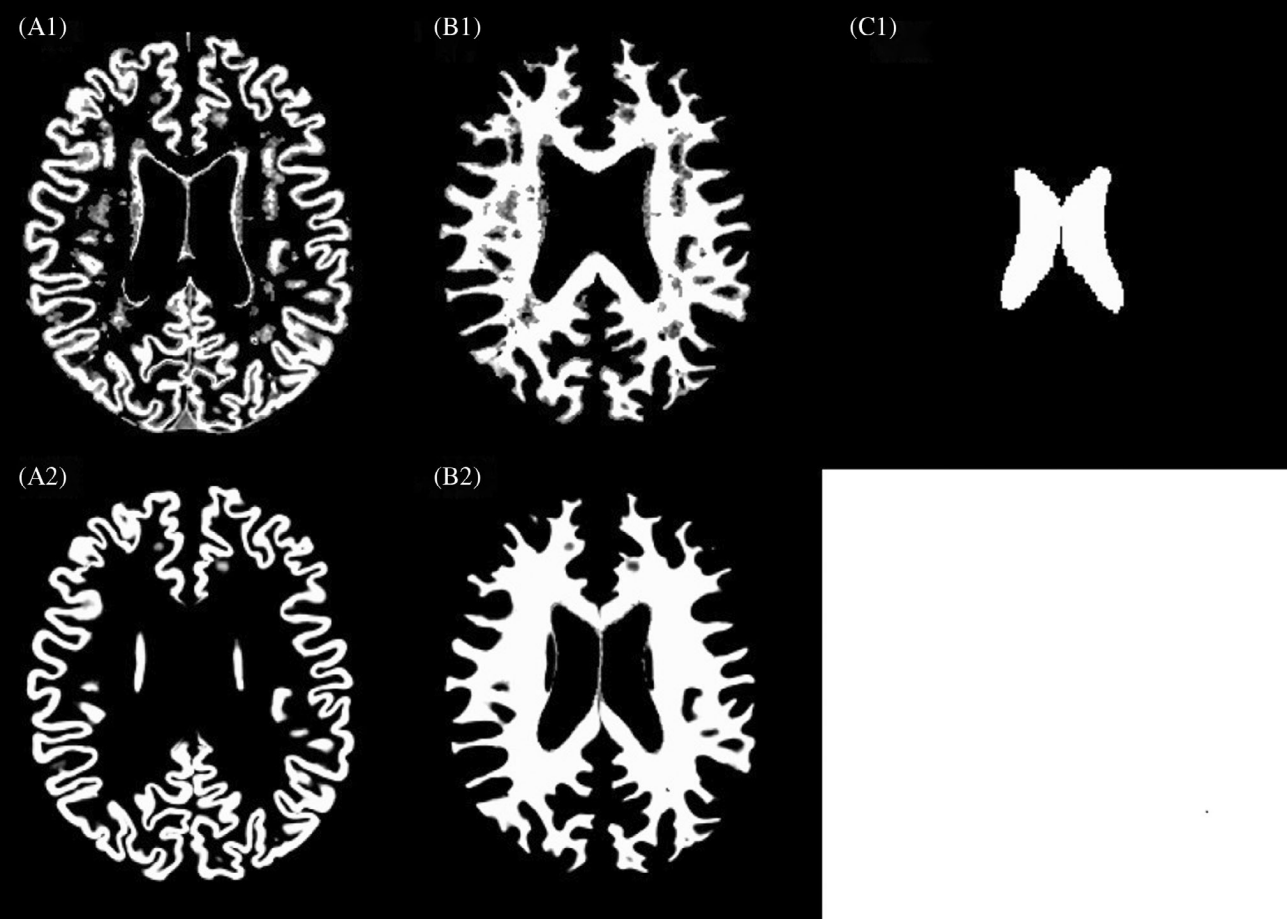
Partial volume estimates from FSL FAST (*top*) cerebrospinal fluid and from Toblerone (*bottom*). 1(a), 2(a): Gray matter. 1(b), 2(b): White matter. 1(c): Cerebrospinal fluid.

To obtain PV estimates, the volumetric pipeline used PV estimates (GM/WM/CSF) calculated by FSL FAST^29^ with the preprocessed T1w image (see Figure 2). The surface‐based pipeline utilized Toblerone to calculate GM/WM PVs, leveraging the structural information provided by *Freesurfer* (see Figure 2). This approach allowed us to estimate PVs within the cortex and all structures identified by *Freesurfer* in the structural space.

#### 2.4.3 ASL image processing

With corrected T_1_‐weighted image and its PV estimates, pCASL images were processed using OXASL (https://github.com/physimals/oxasl), a pipeline for performing Bayesian analysis of ASL‐MRI data, an updated version of the BASIL toolbox found in FSL,^21^ giving access to more advanced regions and surface analysis options. Within the OXASL pipeline, motion correction was first calculated for ASL data using FSL MCFLIRT, with the calibration image as reference. Then the perfusion‐weighted image derived from the ASL data was registered to the corrected T_1_‐weighted image using FSL FLIRT (https://fsl.fmrib.ox.ac.uk/fsl/fslwiki/FLIRT) to obtain the transformation between native space and structural space. B_0_ maps were used to calculate distortion corrections for the ASL data, compensating for any spatially nonlinear image distortions caused by B_0_ inhomogeneities in these EPI data. Sensitivity correction was also applied to account for variable sensitivity of the radiofrequency receive coil being used. At the end of preprocessing, these corrections were applied to ASL data and calibration data. Quantification of the ASL data was conducted in native space using Bayesian inference, following the standard well‐mixed, single‐compartment kinetic model without dispersion of the bolus of labeled blood water.^35^ Assumptions included a tissue T_1_ value of 1300 ms, an arterial blood T_1_ value of 1650 ms,^6^ and a blood–brain partition coefficient of 0.9. The labeling efficiency was set at 0.85.^6^ A longer PLD of 1800 ms was used for superior slices to correct the slice delay of 30 ms. PVEc was performed by BASIL^21^ in OXASL using PV estimates supplied from FAST (volumetric pipeline) or Toblerone (surface‐based pipeline). Voxel‐wise absolute perfusion values (CBF in mL/100 g/min) were subsequently calculated using the calibration data (M_0_),^6^ unlike the previous analysis of this data set which used CSF as a reference region. Finally, regional analysis was carried out for CBF maps in the native space in the volumetric pipeline. A threshold of 80% gray matter PV was used to define “pure” gray matter, where the mean GM CBF was calculated for the non‐PVEc data. For PVEc data, the mean GM CBF was calculated and averaged across all voxels within the GM mask in which there was GM tissue. In the surface‐based pipeline, global CBF values and cortical regional analysis were conducted after mapping the (non‐PVEc and PVEc) CBF maps onto the cortical surface. Notably, zero‐perfusion vertices were excluded and only non‐zero values were calculated for the global and parcels.

#### 2.4.4 Mapping pCASL onto cortical surface

**FIGURE 3:**
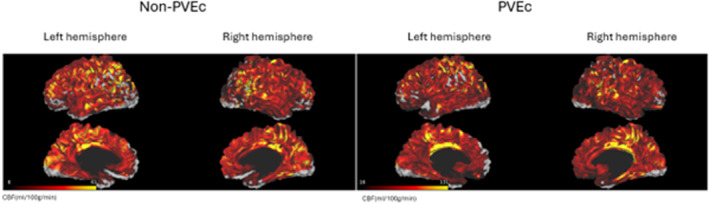
Non–partial volume effect correction (PVEc) arterial spin labeling (ASL) image (*left*) and PVEc ASL image (*right*) from the same subject in the standard space projected onto the 32k_fs_LR cortical midthickness surface. CBF, cerebral blood flow.

To enable surface‐based analyses, this study followed the processing procedure from the HCP functional MRI Surface pipeline.^20^ First, a pCASL image in MNI volume space was mapped onto the midthickness surface in native mesh using the ribbon‐constrained algorithm from the HCP workbench command (volume‐to‐surface‐mapping). A weighted PV map was input to the method to better distinguish the contribution of voxels partially inside or outside the GM ribbon. Ultimately, the cortical CBF map was resampled onto 32k_fs_LR mesh. The non‐PVEc and PVEc ASL images from a participant projected onto midthickness surfaces on 32k_fs_LR mesh in MNI volume space is shown in Figure 3.

### 2.5 Statistical analysis

**FIGURE 4:**
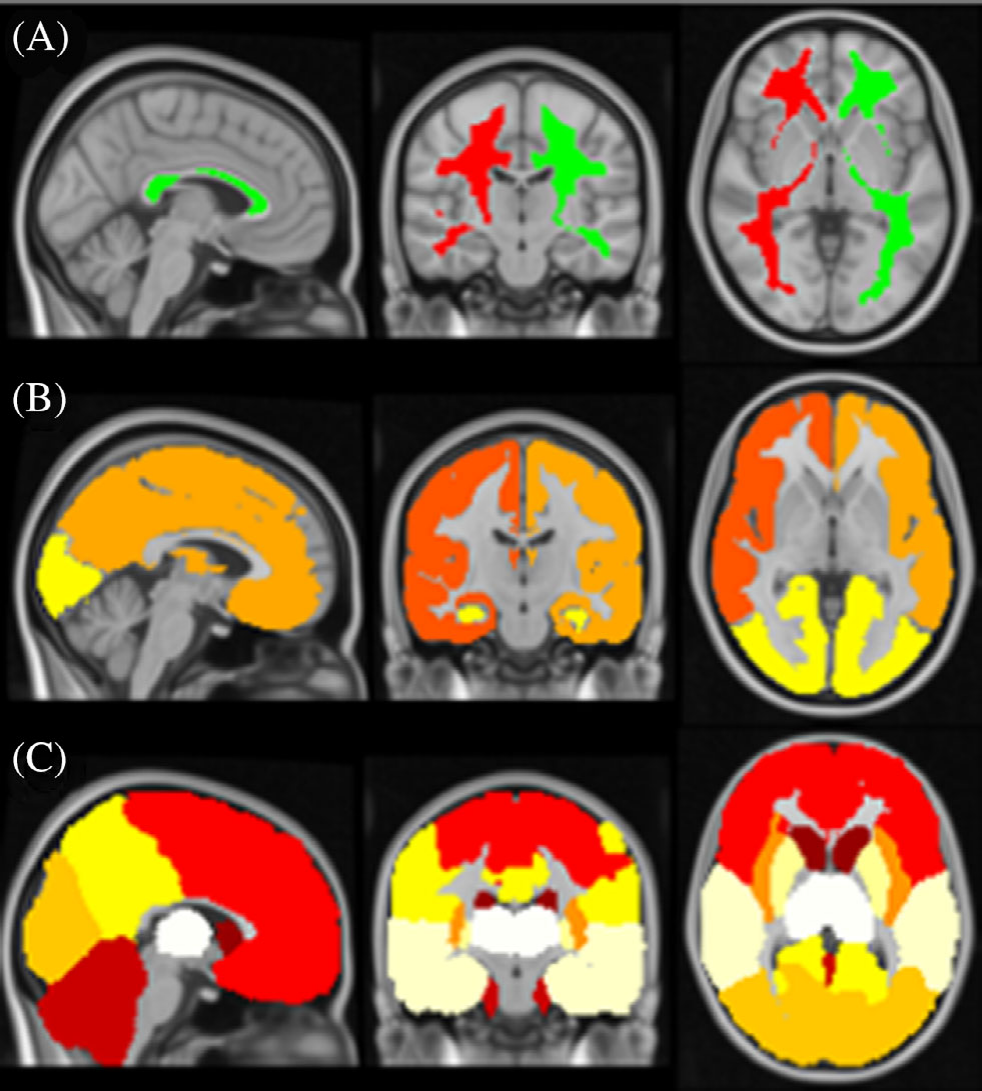
Atlases and regions of interest (ROIs) used in volumetric analysis. (A) Cerebral white matter. (B) Vascular territories (right internal carotid artery territory, left internal carotid artery territory, and vertebrobasilar arteries territory). (C) Eighteen ROIs used in UK Biobank^36^ in the standard space, including caudate, cerebellum, frontal lobe, insula, occipital lobe, parietal lobe, putamen, temporal lobe, and thalamus.

Variations in CBF with age and sex were modeled using linear regression with ordinary least squares using *Python* (version 3.11.8). Age was integrated into the models as a continuous variable, with gender serving as a covariate. Additionally, the models took into account potential interactions between age and sex. Statistical significance was set at *p* < 0.05. In volumetric analysis, linear regressions were built for the whole‐brain GM and for ROIs derived from cerebral WM, vascular territories,^13^ and the brain atlas including 18 regions (see Figure 4) used in UK Biobank imaging study neuroimaging analysis pipeline. In surface‐based analysis, models were calculated for the whole cortex and bilateral 33 cortical parcels from Desikan‐Killiany atlas.^37^ Normative reference values of non‐PVEc and PVEc mean GM CBF are reported for both males and females at 5‐year intervals between the ages of 54 and 84, across the 5th, 10th, 25th, 50th, 75th, 90th, and 95th percentiles.

## 3 RESULTS

For each subject, we obtained the mean and regional CBF values in volumetric regions and cortical parcels from non‐PVEc and PVEc CBF maps.

### 3.1 Volumetric results

#### 3.1.1 Effect of age on global CBF

**FIGURE 5:**
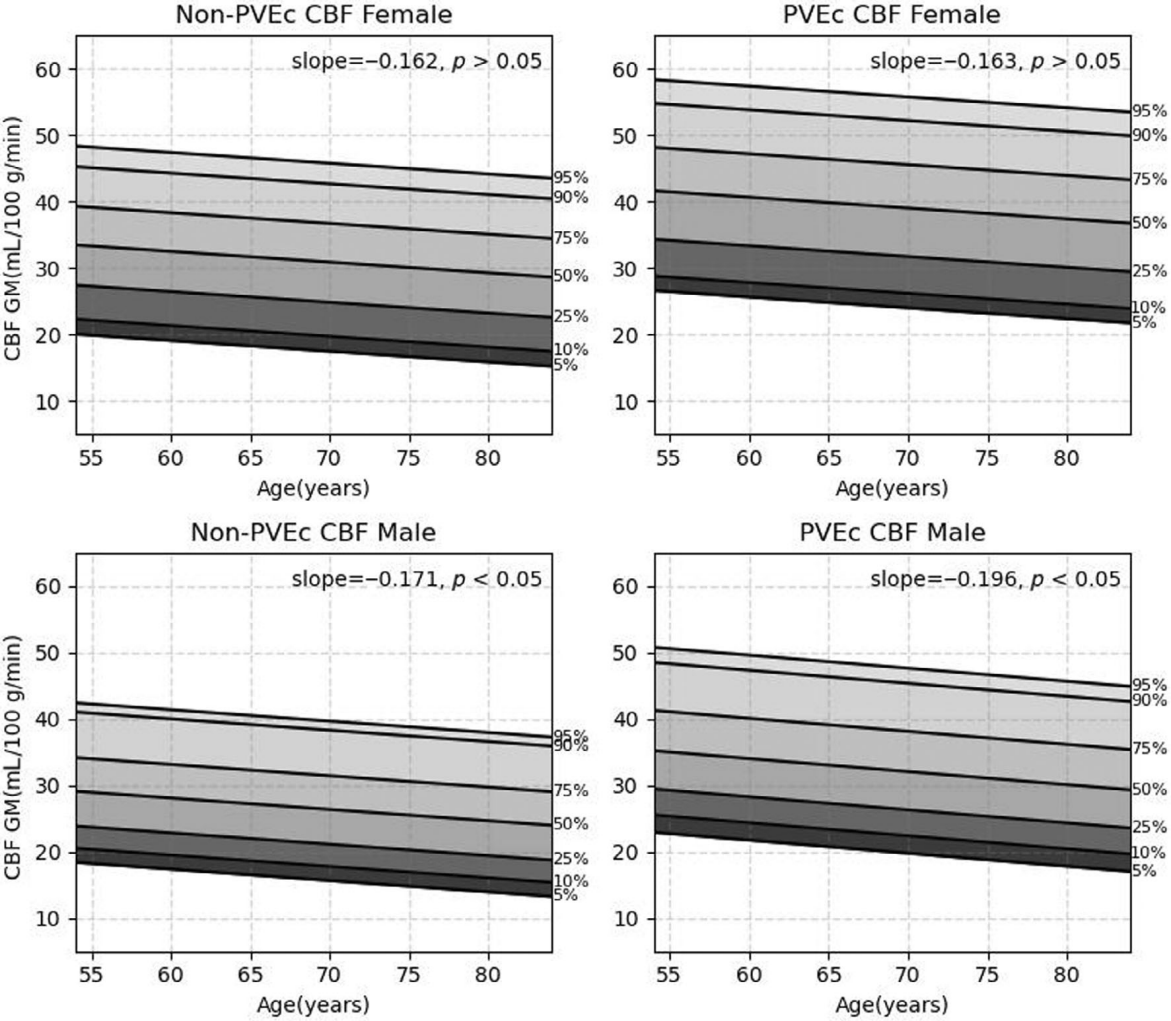
Age‐related gray‐matter (GM) cerebral blood flow (CBF) changes by sex and with/without partial volume effect correction (PVEc). *Top row*: Non‐PVEc results. Bottom row: PVEc results. The age‐related normative values for GM CBF are displayed across the 5th, 10th, 25th, 50th, 75th, 90th, and 95th percentiles from low to high. The decreasing trends were significant in males but not in females.

Age‐related GM CBF changes classified by sex and with/without PVEc are presented in Figure 5. For non‐PVEc ASL data, the mean GM CBF in the brain was 40.66 ± 8.5 mL/100 g/min (range: 14.98–70.97 mL/100 g/min). GM CBF decreased by 0.17 mL/100 g/min for each year of aging (*p* < 0.05) and was on average 3.5 mL/100 g/min higher in females (*p* < 0.01). In males, the decrease in GM CBF with age was significant (*p* < 0.05) and was equivalent to a decrease of 17.1% across the age range tested from 41.21 mL/100 g/min in the youngest (54 years) to 36.08 mL/100 g/min in the oldest (84 years). However, the decline in GM CBF with age in females was not significant.

After PVEc, the mean global GM CBF in the brain was 48.56 ± 9.8 mL/100 g/min (range: 19.28–82.6 mL/100 g/min). GM CBF decreased by 0.18 mL/100 g/min for each year of aging (*p* < 0.05) and was 3.8 mL/100 g/min higher in females (*p* < 0.05). In males, the decrease in GM CBF with age was significant (*p* < 0.05) and was equivalent to a decrease of 19.6% across the tested age range from 49.32 mL/100 g/min to 43.43 mL/100 g/min. No significant variation was observed in females.

#### 3.1.2 Effect of age on regional CBF

Age‐related CBF variations in volumetric ROIs from non‐PVEc and PVEc data are given in Table 2. Slopes of significance are marked with asterisks. In most significant regions, CBF exhibited a decreasing trend, but an increase was only found in the left putamen in PVEc data.

**TABLE 2:** Age‐related CBF variations in ROIs from the volumetric pipeline for non‐PVEc and PVEc ASL.

ROI	Non‐PVEc			PVEc		
	Slope	GM CBF	Sex difference	Slope	GM CBF	Sex difference
LICA	−0.12*	36.56	−4.25	−0.05	82.53	−4.4
RICA	−0.16**	40.28	−3.47	−0.17	83.97	−4.07
VBA	−0.09	40.18	−3.31	0.05	65.45	−3.71
Left cerebral white matter	−0.05	20.59	−2.5	−0.32**	63.8	−3.18
Right cerebral white matter	−0.07*	19.96	−2.38	−0.39**	62.57	−3
Left caudate	−0.31**	24.17	−3.97	0.09	52.4	−3.23
Left cerebellum	−0.06	31.35	−3.38	0.12	51.85	−2.92
Left frontal lobe	−0.07	37.04	−2.96	−0.11	86.02	−4.79
Left insula	−0.18**	36.63	−2.32	−0.23*	65.1	−1.96
Left occipital lobe	−0.09	35.62	−3.88	0.13	72.69	−3.67
Left parietal lobe	−0.12*	36.15	−3.59	−0.08	89.02	−5.71
Left putamen	0.09*	36.49	−1.57	0.24*	64.93	−0.5
Left temporal lobe	−0.08	32.85	−3.03	0.03	59.84	−3.32
Left thalamus	−0.03	34.94	−4.27	0	82.29	−7.95
Right caudate	−0.34**	23.55	−4.12	0.08	51.55	−3.45
Right cerebellum	−0.06	31.18	−3.53	0.08	51.98	−3.23
Right frontal lobe	−0.12*	36.79	−2.95	−0.23	85.6	−4.6
Right insula	−0.22**	35.89	−2.41	−0.30*	65.16	−2.46
Right occipital lobe	−0.1	36.56	−4.14	0.05	76.89	−4.75
Right parietal lobe	−0.13*	36.64	−3.38	−0.21	93.36	−5.44
Right putamen	0.02	36.22	−1.57	0.1	65.66	0.33
Right temporal lobe	−0.1*	32.77	−2.61	−0.04	62.8	−2.45
Right thalamus	−0.03	34.52	−4.29	0.06	81.52	−8.49

*Note*: Slope unit: mL/100 g/min per year. CBF gray‐matter unit: mL/100 g/min. Sex difference (male–female) unit: mL/100 g/min.

Abbreviations: ASL, arterial spin labeling; CBF, cerebral blood flow; LICA, left internal carotid artery territory; RICA, right internal carotid artery territory; ROI, region of interest; PVEc, partial volume effect correction; VBA, vertebrobasilar arteries territory.

* *p* < 0.05.

** *p* < 0.01.

### 3.2 Surface‐based results

#### 3.2.1 Effect of age on global cortical CBF

For non‐PVEc CBF, the mean cortical GM CBF was 35.60 ± 7.34 mL/100 g/min (range: 19.1–47.5 mL/100 g/min). GM CBF decreased by 0.22 mL/100 g/min for each year of aging (*p* < 0.01) and was on average 3.44 mL/100 g/min higher in females (*p* < 0.01). For PVEc ASL data, the mean cortical GM CBF was 67.09 ± 13.42 mL/100 g/min (range: 27.1–89.4 mL/100 g/min). GM CBF decreased by 0.05 mL/100 g/min for each year of aging (*p* > 0.1) and was on average 3.67 mL/100 g/min higher in females (*p* < 0.01).

#### 3.2.2 Effect of age on regional cortical CBF

Table 3 lists the regional CBF‐GM variations with age on non‐PVEc and PVEc data in cortical bilateral parcels from the Desikan‐Killiany atlas before and after PVEc over all subjects. Before PVEc, parcels with significant GM‐CBF variations all exhibited decreases with age. After PVEc, the decreasing trend of GM CBF with age was no longer statistically significant in most parcels. Only seven parcels (caudal anterior cingulate, caudal middle frontal, pars opercularis, rostral anterior cingulate, superior frontal, superior parietal, and insula) still exhibited significant reduction with age, whereas significantly increasing CBF with age was found in the bank's superior temporal, fusiform, and inferior temporal regions.

**TABLE 3:** The cortical GM‐CBF variations with age, with and without PVEc, in Desikan‐Killiany atlas over all subjects.

ROI	Non‐PVEc			PVEc		
	Slope	GM CBF	Sex difference	Slope	GM CBF	Sex difference
Banks superior temporal sulcus	−0.17**	42.07	−4.14	0.32**	77.88	−1.08
Caudal anterior‐cingulate cortex	−0.46**	29.93	−3.14	−0.3**	65.53	−2.74
Caudal middle frontal gyrus	−0.26**	44.38	−4.5	−0.21*	87.99	−5.56
Cuneus cortex	−0.24**	42.55	−4.95	0.1	74.26	−5.21
Entorhinal cortex	−0.06	20.11	−1.73	−0.04	36.7	−5.08
Fusiform gyrus	−0.06	27.18	−5.52	0.14*	53.33	−10.46
Inferior parietal cortex	−0.31**	43.1	−4.38	0.08	81.37	−1.23
Inferior temporal gyrus	−0.07	23.44	−4.66	0.12*	44.64	−5.47
Insula	−0.28**	36.01	−2.6	−0.29**	59.5	−2.75
Isthmus‐cingulate cortex	−0.29**	37.8	−3.29	0	80.83	−7.53
Lateral occipital cortex	−0.25**	32.14	−6.65	−0.1	64.45	−7.77
Lateral orbital frontal cortex	−0.07*	27.14	−2.16	0.02	46.28	−2.71
Lingual gyrus	−0.12**	35.48	−5.05	0.08	68.74	−11.37
Medial orbital frontal cortex	−0.19**	29.08	−2.56	−0.08	44.18	−2.67
Middle temporal gyrus	−0.14**	37.19	−3.81	0.12	62.72	−2.92
Paracentral lobule	−0.16**	42.45	−3.24	0.07	81.99	−3.31
Parahippocampal gyrus	−0.02	28.06	−4.11	0.11	52.79	−8.05
Pars opercularis	−0.28**	41.68	−2.98	−0.16*	74.12	−2.95
Pars orbitalis	−0.06	23.41	−0.59	0.09	45.83	−0.91
Pars triangularis	−0.24**	38.4	−2.37	−0.07	66.17	−2.57
Pericalcarine cortex	−0.17**	41	−4.46	0.07	74.82	−4.82
Postcentral gyrus	−0.2**	42.45	−3.35	0	83.53	−3.66
Posterior‐cingulate cortex	−0.49**	35.6	−2.25	−0.13	78.96	−2.68
Precentral gyrus	−0.17**	42.48	−2.75	0	85.72	−2.41
Precuneus cortex	−0.28**	42.62	−3.89	−0.01	77.61	−4.38
Rostral anterior cingulate cortex	−0.43**	39.39	−3.49	−0.21**	62.49	−4.09
Rostral middle frontal gyrus	−0.17**	39.58	−3.21	−0.03	67.2	−2.35
Superior frontal gyrus	−0.21**	39.44	−3.34	−0.18*	72.78	−4.18
Superior parietal cortex	−0.33**	38.98	−5.32	−0.22*	82.33	−5.4
Superior temporal gyrus	−0.2**	39.51	−2.56	0.07	69.61	−2.1
Supramarginal gyrus	−0.27**	41.45	−3.11	−0.01	77.33	−2.24
Temporal pole	−0.1*	19.23	−1.7	−0.02	31.21	−5.27
Transverse temporal cortex	−0.3**	48.44	−1.21	0.08	91.03	0.7

*Note*: Slope unit: mL/100 g/min per year. GM‐CBF unit: mL/100 g/min. Sex difference (male–female) unit: mL/100 g/min.

Abbreviations: CBF, cerebral blood flow; GM, gray matter; PVEc, partial volume effect correction; ROI, region of interest.

* *p* < 0.05.

** *p* < 0.01.

## 4 DISCUSSION

This study investigated the regional changes in perfusion with age and has explored the influence of changes in anatomy with age on these observations by attempting to correct for variation in GM volume. To our knowledge, this is the first study using both volumetric and surface‐based analyses with the correction of PVEs with ASL‐MRI perfusion in a large cohort.

Perfusion images, especially ASL, are conventionally analyzed using the volumetric method, which is susceptible to PVEs. In this study, we mapped the ASL image onto the cortical surface to provide a better representation of cortical GM anatomy.^27^, ^30^ Furthermore, we compared PVEc and non‐PVEc results, following the suggestions from Chappell et al.,^15^, ^16^ specifically to explore the role of PVEc and thus control for apparent changes in perfusion that are actually due to alterations in anatomy.

The former study on the TILDA ASL data^28^ reported a mean global GM CBF (36.5 ± 8.2 mL/100 g/min) and decreased by 0.2 mL/100 g/min per year without PVEc. In contrast, this study found a slightly higher mean GM CBF in the whole brain (40.66 ± 8.5 mL/100 g/min) and a lower CBF decreasing slope by 0.17 mL/100 g/min per year before PVEc. The reason for the observed differences could largely be due to the different calibration methods used and the use of voxel‐wise calibration replacing the CSF reference region calibration in the former study. Our results are consistent with the recommendations of Pinto et al.,^6^ who higher CBF values in voxel‐wise calibration compared with CSF reference region using single‐PLD pCASL data.^38^ Prior research has used a range of perfusion imaging techniques to explore normative non‐PVEc GM CBF values for different age groups. A study by Jefferson et al., following a similar protocol as ours, reported mean whole‐brain CBF values of 37.3 ± 7.1 mL/100 g/min for 270 adults with an average age of 73 ± 7 years.^39^ In a smaller study with different age groups using 3T pulsed‐ASL MRI, Chen et al. reported higher mean cortical CBF values of 52.6 ± 9.3, 52 ± 10.7, and 42.7 ± 8.8 mL/100 g/min in young, middle‐aged, and older groups, respectively.^7^ Biagi et al., using continuous ASL, found a mean GM CBF of 58.4 mL/ 100 g/min for the 21 adults (mean age 40 ± 15 years).^40^ After PVEc, the mean GM CBF was found to be higher compared with non‐PVEc GM CBF, which was consistent with previous comparisons with and without PVEc.^14^, ^41^ Few existing studies have reported global perfusion with PVEc. For instance, a study by Meltzer et al. reported mean cortical CBF using positron emission tomography for younger (62 ± 10 mL/100 g/min) and older (62 ± 10 mL/100 g/min) groups with PVEc, which was close to our results despite the use of a different perfusion technique.^42^ Preibisch et al., using pulsed‐ASL MRI, reported global GM‐CBF values of 40.9 ± 5.5 mL/100 g/min and 42.0 ± 8.6 mL/100 g/min for 19 young and 25 older adults, respectively, with PVEc.^11^

We found a global decrease of GM CBF with age before PVEc in both volumetric and surface‐based analysis, and the slopes were not statistically different when evaluated by t‐test. After PVEc, we found a greater decline of GM CBF with aging in volumetric analysis, although the slope was not statistically significantly different from that found before PVEc. Because post–PVEc‐GM perfusion values were large than precorrection, the greater slope observed might simply be due to this reason; hence, we also calculated the slope scaled by the mean GM CBF before and after PVEc, and we found the former was bigger than the latter (ratio between them 1.11). The relative reduction in the scaled slope values would be consistent with PVEc removing a component of apparent perfusion reduction with age, which is associated with PVEs and changes in brain structure. These results would tend to support the view that there is a genuine decrease in perfusion in GM with age.^7^, ^8^, ^9^

After PVEc, the surface‐based analysis indicated a negligible change in CBF with aging: A small decreasing slope was observed, but this was not statistically significant. This potentially contradicts the volumetric results; however, it might be a result of the acquisition and analysis used in this case. Notably, the data have a relatively large slice thickness compared with cortical thickness, and PV correction was performed in volumetric space before projection onto the surface, all of which makes the surface‐based study of these data exploratory and worth interpreting with care. Data with higher resolution and PV correction optimized for surface analysis will need be adopted in future work to examine whether these findings generalize.

Regarding regional age‐related GM‐CBF variations in our study, in the volumetric analysis, we found regional GM‐CBF reductions with age in most ROIs before PVEc; in some ROIs, this was no longer significant after PVEc. In surface‐based analyses, the regional cortical GM‐CBF values were found to decrease with age before PVEc. After PVEc, seven parcels remain a decreasing trend, with three parcels showing an increase in perfusion. Previous studies have also investigated regional CBF changes with age with and without PVEc.^7^, ^43^, ^44^, ^45^, ^46^, ^47^ For example, Parkes et al. observed that age‐related changes in GM without PVE correction were predominantly localized in the frontal cortex using CASL MRI.^47^ Martin et al. found that non‐PVEc CBF values decreased with age in several regions, including the cingulate, parahippocampal, superior temporal, medial frontal, and posterior parietal cortices bilaterally, as well as in the left insular and left posterior prefrontal cortices.^48^ Lee et al. identified both decreased and increased regional non‐PVEc CBF values, with the most common regions for decreased perfusion being the precuneus, superior temporal, and orbitofrontal, and for increased perfusion, the caudate, posterior cingulate, anterior cingulate, and amygdala.^12^ Parkes et al. detected age‐related non‐PVEc GM‐CBF reduction in the anterolateral prefrontal cortex and in areas along the lateral sulcus and the lateral ventricle, bilaterally.^47^ Zhang et al. observed that CBF without PVEc using pCASL demonstrated decreases with age in the frontal and parietal regions and the cerebellum, alongside increases in the temporal and occipital areas.^49^ Preibisch et al. using PASL MRI reported age‐related CBF decreases without PVEc in frontal, parietal, insular, cingulate, parahippocampal, and caudate. Few prior studies reported cortical GM CBF with PVEc. For example, Preibisch et al. reported decreases in GM CBF after PVEc in the parietal cortex, cuneus, and caudate.^11^ Furthermore, Preibisch et al. also observed increases of CBF after PVEc, similar to ours, in the lateral and medial temporal lobe such as hippocampus, the calcarine gyrus, and the thalamus.^11^

Previous studies have also highlighted a dissociation between regional CBF and structural alterations specific to normal aging. Moreover, they suggest that other factors might influence age‐related perfusion changes. For instance, Chen et al. reported that regions experiencing CBF reduction are largely distinct from those most affected by GM atrophy, indicating that hemodynamic and anatomical changes may differentially contribute to age‐related cognitive decline.^7^ In the study by Parkes et al., they suggest that, without significant medical conditions, healthy aging might not affect resting cortical perfusion.^47^ Other research indicates that the observed reduction in perfusion could be attributed to progressive neuronal loss, reduced neuronal activity, and a decline in the synaptic density of brain neurons.^50^, ^51^ This study provides further evidence for regionally specific perfusion changes with age that are potentially independent of anatomical changes, but cannot further explain what the mechanisms behind this might be.

We also investigated sex differences in age‐related CBF changes and found females exhibited higher global GM‐CBF values compared with males before and after PVEc (*p* < 0.01), which was consistent with some other studies.^52^, ^53^ Moreover, previous studies have investigated the rate of global CBF decline between males and females with respect to normal aging.^53^, ^54^ However, we cannot reach a related conclusion, as the variations of global CBF with age in females was not statistically significant.

One limitation regarding the pCASL‐MRI protocol is that it applied the same labeling duration and delay for all participants, effectively presuming negligible impact from possible spatial variations in arterial transit time—the time it takes for the arterial blood bolus to travel from the labeling plane to the imaging voxels. However, arterial transit time can vary regionally between individuals due to different age groups, health conditions, and populations.^6^ A PLD of 1800 ms was considered the optimal choice among our cohort with a mean age of less than 70 years, to obtain adequate tissue signals and to minimize intravascular signals.^55^ Consequently, this could lead to potential underestimation or overestimation of CBF measurements across the cohort of a wide age range (54–84 years) in this study.

The 8‐mm slice thickness of ASL acquisitions was substantially large for attempting surface mapping, and this may have led to the differences observed in changes in regional perfusion with age from both pipelines, noting also that regions defined in volumetric space and on the surface do not have a direct correspondence. Moreover, PVEs were corrected in the volumetric space in this study, although correction for PVEs directly in the surface space would potentially be superior. However, the field still lacks the necessary algorithms to fully implement this approach. The use of surface‐based representations for ASL data is still in its infancy, and this study at least suggests more work might be required to handle PVE‐related effects when performing surface projection.

Small patches of zero perfusion of CBF map were spotted on the cortex (Figure 3). The reason may be that the cortex of the CBF map was not perfectly aligned with the template surface, as registrations of the CBF map (from native space to structural space and finally to the standard space) were conducted using volumetric methods (i.e., linear and nonlinear registrations)—or the deficiency of CBF map itself. Furthermore, extremely high perfusion of some vertices was found on the cortex, which may be related to macrovascular contamination that cannot be corrected for in the single‐PLD ASL data. It is worth noting that cortical perfusion could fluctuate slightly if resampled on a cortical mesh of different resolutions due to interpolation; thus, we made efforts to avoid redundant registration/smoothing as much as possible.

For ROI analyses, the influences of age and sex on CBF were investigated using linear regression in 35 volumetric ROIs and on 33 bilateral cortical parcels. The threshold for statistical significance level was *p* < 0.05 without the correction of multiple comparisons; however, this will increase the risk of Type I errors and false discovery rate, given the number of regions reported and different analysis approaches adopted in this study. Because this study was exploratory, we have not implemented multiple comparison corrections and have simply reported all results as a reference for other work. Future studies will be required with focused hypotheses to draw firm conclusions about changes in specific brain regions.

## 5 CONCLUSION

A volumetric pipeline and a surface‐based pipeline were applied in this study for pCASL data. With PVE correction applied, we found a global GM‐CBF reduction with aging before PVEc but regionally selective after PVEc.

## CONFLICT OF INTEREST

Michael A. Chappell receives royalties for commercial licensing of FSL. Michael A. Chappell and Martin S. Craig are employed by Quantified Imaging Ltd.

## Data Availability

The data sets are not currently available publicly due to data‐protection regulations but are accessible from TILDA on reasonable request at https://tilda.tcd.ie/data/accessing‐data/. MRI analysis was performed using the Human Connectome Project pipeline, and the workbench can be found at https://www.humanconnectome.org, oxford_asl in the FMRIB Software Library (BASIL–FSL: https://fsl.fmrib.ox.ac.uk/fsl/fslwiki/BASIL), oxasl at https://github.com/ibme‐qubic/oxasl, and Toblerone at https://github.com/tomfrankkirk/toblerone. The scripts for processing and analysis can be found at https://github.com/physimals/tilda_asl.
